# Six-month follow-up after recovery of COVID-19 Delta variant survivors *via* CT-based deep learning

**DOI:** 10.3389/fmed.2023.1103559

**Published:** 2023-02-02

**Authors:** Jianliang Huang, Ruikai Lin, Na Bai, Zhongrui Su, Mingxin Zhu, Han Li, Conghai Chai, Mingkai Xia, Ziwei Shu, Zhaowen Qiu, Mingsheng Lei

**Affiliations:** ^1^Zhangjiajie Hospital Affiliated to Hunan Normal University, Zhangjiajie, China; ^2^College of Information and Computer Engineering, Northeast Forestry University, Harbin, China; ^3^Yong Loo Lin School of Medicine, National University of Singapore, Singapore, Singapore; ^4^Heilongjiang Tuomeng Technology Co., Ltd., Harbin, China; ^5^Zhangjiajie College, Zhangjiajie, China

**Keywords:** follow-up, Delta variant survivors, deep lung parenchyma enhancing, sub-visual lesion, pulmonary fibrosis, COVID-19 sequelae

## Abstract

**Purpose:**

Using computer-aided diagnosis (CAD) methods to analyze the discharge and 6-month follow-up data of COVID-19 Delta variant survivors, evaluate and summarize the recovery and prognosis, and improve people's awareness of this disease.

**Methods:**

This study collected clinical data, SGRQ questionnaire results, and lung CT scans (at both discharge and 6-month follow-up) from 41 COVID-19 Delta variant survivors. Two senior radiologists evaluated the CT scans before in-depth analysis. Deep lung parenchyma enhancing (DLPE) method was used to accurately segment conventional lesions and sub-visual lesions in CT images, and then quantitatively analyze lung injury and recovery. Patient recovery was also measured using the SGRQ questionnaire. The follow-up examination results from this study were combined with those of the original COVID-19 for further comparison.

**Results:**

The participants include 13 males (31.7%) and 28 females (68.3%), with an average age of 42.2 ± 17.7 years and an average BMI of 25.2 ± 4.4 kg/m^2^. Compared discharged CT and follow-up CT, 48.8% of survivors had pulmonary fibrosis, mainly including irregular lines (34.1%), punctuate calcification (12.2%) and nodules (12.2%). Compared with discharged CT, the ground-glass opacity basically dissipates at follow-up. The mean SGRQ score was 0.041 (0–0.104). The sequelae of survivors mainly included impaired sleep quality (17.1%), memory decline (26.8%), and anxiety (21.9%). After DLPE process, the lesion volume ratio decreased from 0.0018 (0.0003, 0.0353) at discharge to 0.0004 (0, 0.0032) at follow-up, *p* < 0.05, and the absorption ratio of lesion was 0.7147 (–1.0303, 0.9945).

**Conclusion:**

The ground-glass opacity of survivors had dissipated when they were discharged from hospital, and a little fibrosis was seen in CT after 6-month, mainly manifested as irregular lines, punctuate calcification and nodules. After DLPE and quantitative calculations, we found that the degree of fibrosis in the lungs of most survivors was mild, which basically did not affect lung function. However, there are a small number of patients with unabsorbed or increased fibrosis. Survivors mainly had non-pulmonary sequelae such as impaired sleep quality and memory decline. Pulmonary prognosis of Delta variant patients was better than original COVID-19, with fewer and milder sequelae.

## 1. Introduction

Since first detected in Wuhan, China, Coronavirus disease 2019 (COVID-19) has swept the world, threatening the world with public health concerns and social instability. As of 3rd November 2022, the cumulative number of confirmed COVID-19 cases worldwide reached 631,324,387, with more than 6,594,803 cumulative deaths (1). The major pathogen of COVID-19 has been identified as severe acute respiratory syndrome coronavirus 2 (SARS-CoV-2), yet new variants kept appearing, leading to ongoing worldwide outbreaks of COVID-19 at different magnitudes. SARS-CoV-2 Delta variant (also known as lineage B.1.617.2), a variant of concern identified by the World Health Organization (WHO), became the primary strain of the COVID-19 pandemic in 2021 (2), affecting more than 75% of countries worldwide. In March 2022, a new variant named Deltacron with Delta variant as the main stem was confirmed to exist by WHO, which will exacerbate the plague of COVID-19 to humans. Therefore, it is very important and urgent to fully understand COVID-19, especially the mechanism of action and physiological effects of SARS-CoV-2 and its variants on humans.

In the mid-1960s, Lodwick first introduced the concept of using computer technology for medical image analysis and computer-aided diagnosis (CAD). However, limitations such as technology and clinical philosophy have constrained the development of CAD technology. It was not until after the 1980s, with the development of mathematics, statistics, data mining techniques, computer algorithms and other sciences, that CAD emerged in large numbers in the treatment and prognosis studies of many diseases (3). Notably, the rapid development of artificial intelligence (AI) has surged the recent CAD craze, enabling the application of technologies such as machine learning and deep learning in clinical diagnosis, treatment, and prognosis. To date, AI has gradually emerged in various medical fields and clinical challenges, such as tumor diagnosis, cardiovascular diseases, and central nervous system pathologies (4, 5). During the COVID-19 pandemic, AI approaches have been extended to understanding COVID-19 pneumonia from multiple perspectives, including prevention, diagnosis, treatment, monitoring, and follow-up examination, as such to provide an abundance of valuable clinical evidence and decision support for fighting against the disease (6).

We collected academic research on COVID-19 (SARS-CoV-2 virus) and its variants from three literature databases, the Web of Science, PubMed, and China National Knowledge Infrastructure, bringing the total number of relevant publications to 640,333 from the earliest searchable date to May 2022. While 19,873 cases were related to follow-up examination, only 101 were associated with the Delta variant. In this study, we followed up with 41 Delta variant survivors from Zhangjiajie City, China, for 6 months after discharge. We collected these patients' last CT scan and clinical data before they were discharged from the hospital and continued to collect CT scans and important clinical indicators during the 6-month follow-up. Further, we used AI approaches such as deep lung parenchyma enhancing (DLPE) to quantify follow-up CT and discharged CT (7) and to provide a comprehensive assessment of patient recovery and prognosis. Our findings provide intrinsic insights into the mechanisms underlying the prognosis of COVID-19, especially the Delta variant.

## 2. Materials and methods

### 2.1. Study design and participants

This is a retrospective study. We collected 6-month follow-up data from COVID-19 Delta variant patients admitted to Zhangjiajie City People's Hospital from July to September 2021. All diagnoses and discharges of patients conformed to the Diagnosis and Treatment of Novel Coronavirus Infection Guidelines produced by the Chinese National Health Commission (Trial Version eight or earlier versions) (8). We excluded the following patients: 1) patients who died before follow-up; 2) patients who refused to participate in follow-up; 3) patients who could not be contacted or otherwise could not participate in follow-up; 4) patients diagnosed with asymptomatic infection at discharge. A total of 41 individuals, including 13 males and 28 females, participated in this follow-up study. We classified the patients into three age groups: youngth (under 45 years), middle-aged (45–59 years), and elderly (60–89 years) in light of the WHO age classification criteria (9). Each individual' s CT scans (at discharge and follow-up) and Body Mass Index (BMI) were collected accordingly. The Chinese BMI standard defines four categories: BMI <18.4 indicates a thin body shape, 18.5 <BMI <23.9 indicates a normal body shape, 24.0 <BMI <27.9 indicates an overweight body shape, and BMI>28.0 indicates an obese body shape. Other clinical data including vaccination status at discharge and SGRQ scores at follow-up were recorded for analysis. Patients with no <1 dose of vaccination history were included in the vaccination cohort concerning the low availability of the COVID-19 vaccine during the Delta variant outbreak in Zhangjiajie. We conducted the hospital discharge and the follow-up CTs with the TOSHIBA Aquilion Lightning CT scanner. The tube voltage and current were set at 120 kV and 100–200 mA, respectively, with a matrix of 512 × 512. Further, we collected the lung window level. The lung window was reconstructed with a 1 mm thin layer, and the scanned lung window level and width were 600 and 1,600 HU, respectively. This retrospective study was approved by the Ethics Committee of the Zhangjiajie City People's Hospital with waived informed consent requirement.

### 2.2. Follow-up assessment

The St. George's Respiratory Questionnaire (SGRQ) (10) is a clinical measurement designed to conduct health status self-assessments for patients with chronic airflow limitation, i.e., various respiratory diseases correlated with pulmonary function (11, 12). The questionnaire contains three main sections: symptoms (respiratory discomfort), activities (impact of dyspnea on daily tasks), and psychosocial impact (psychosocial impact of the disease). Typical SGRQ scores are <1, while higher scores indicate poorer health status and more impaired pulmonary function. Only SGRQ questionnaires filled out by patients without prompting from physicians were used in this study.

Two senior radiologists in the team performed diagnosis on the follow-up and hospital discharge CT scans collected from the 41 COVID-19 Delta variant survivors. We investigated imaging features until consensuses were reached on all diagnostic findings. Further, we categorized all CT scans into two groups: Normal and Abnormal CT (including fibrotic and Non-fibrotic changes). Fibrotic changes include bronchiectasis, reticulations, nodules, punctuate calcification, irregular lines, and pulmonary bullae. Non-fibrotic changes include ground-glass opacity (GGO) and consolidation.

### 2.3. Computer-aided diagnosis

#### 2.3.1. Lesions segmentation of COVID-19

Since the outbreak of COVID-19, a large number of researchers working in artificial intelligence have used deep learning models to assist in the diagnosis, treatment, and prognosis of COVID-19. Their initial goal was generally to save radiologists' time in reviewing medical images and to improve the accuracy of lesions identification. With computer-aided diagnosis methods, physicians can accurately obtain inflammation annotation in CT slices and accurately calculate the percentage of inflammation (POI) and its inflammatory density for each lung lobe or lung segment in a short time (2).

**Figure 1:**
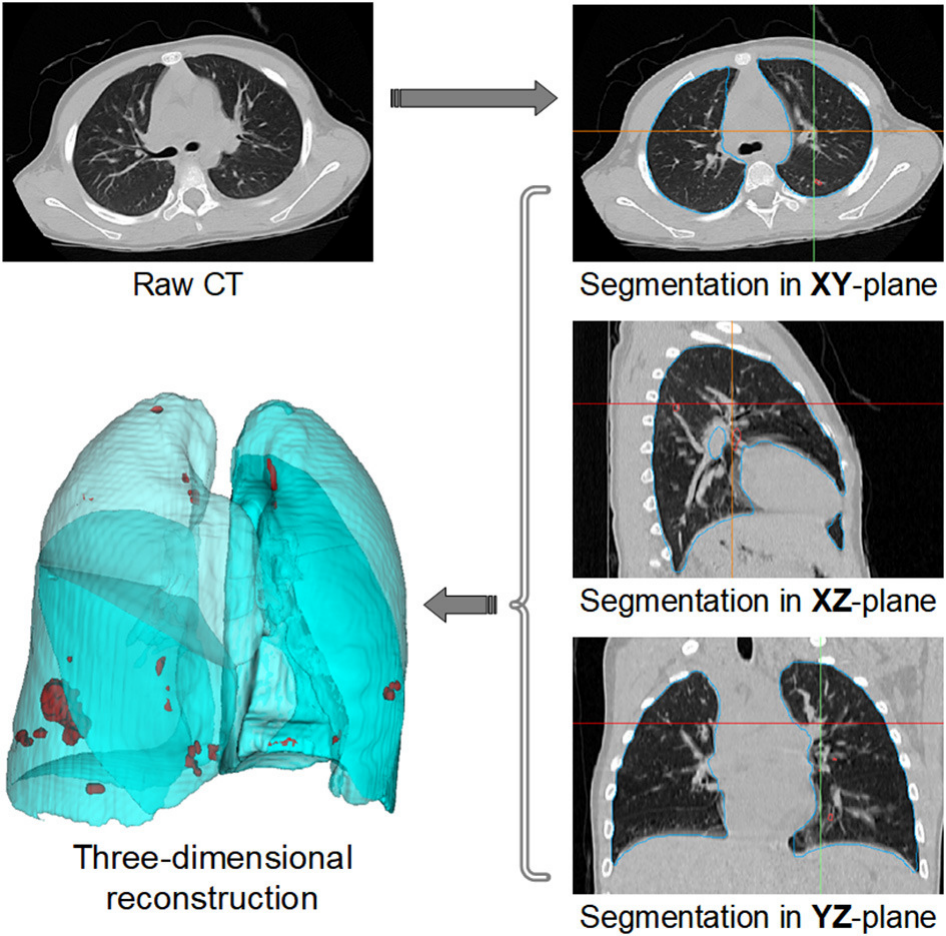
Workflow of traditional lesions segmentation.

A typical workflow is shown in Figure 1. Firstly, raw CT scan is used as input for spatial normalization and signal normalization, and put into the standard space. In the standard space, the inflammation annotation is obtained using our 2.5D segmentation algorithm, i.e., the 3D data is split from three orthogonal directions (XY plane, XZ plane, YZ plane), and the segmentation is performed in each of these three directions using the U-Net network, and then the segmented results are integrated to obtain the final inflammation annotated mask. To better observe the distribution of inflammation in the lung, the results of the inflammation labeling are reconstructed in three dimensions, showing the distribution of inflammation in the lung in a clear and three-dimensional manner. This model can be deployed on an ordinary home computer to mark the inflammation and calculate the POI value of a CT scan within 1 min with the accuracy of more than 97%, which greatly improves the working efficiency of radiologists.

There are certain limitations to such an approach. Namely, the workflow of this model only allows marking visual lesions on regular CT scans, but not sub-visual lesions (i.e., it is almost impossible for a radiologist to see fibrosis lesions directly from ordinary CT scans). Among our team's latest published techniques (7), the deep lung parenchyma enhancing (DLPE) method was used to automatically mark visible and sub-visual COVID-19 lesions. In the follow-up study, we used the DLPE method to avoid the lesion-omissions issue that might occur in similar studies with traditional AI applications.studies.

#### 2.3.2. Deep lung parenchyma enhancing

**Figure 2:**
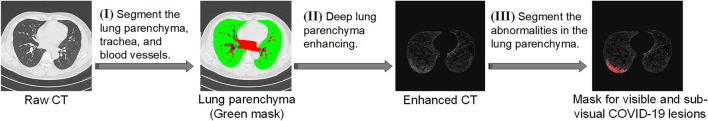
Workflow of deep lung parenchyma enhancing.

Deep lung parenchyma enhancing (DLPE) is a computer-aided detection (CADe) method for quantifying lung parenchymal lesions on chest CT. It can identify new lesions under the original lung window of hospitalized COVID-19 patients and survivors, whereas ordinary CT scans might neglect the sub-visual lesions. DLPE has a solid ability to predict sequelae such as pulmonary fibrosis. Its workflow includes three steps (shown in Figure 2):

(I) Segment the lung parenchyma, trachea and biood vessels. First, we used the proposed 2.5D segmentation algorithm to segment the lung. We further investigated the characteristics of the trachea and blood vessels and developed a two-stage segmentation model accordingly. The first stage determines the approximate extent of the trachea and blood vessels, reducing the search space by thousands of times, upon which the second stage achieves segmentation with higher stability and accuracy. Both stages were carried out by 2.5D segmentation models with feature-enhanced loss function. Finally, we developed a refined trachea and vascular mask.

(II) Deep lung parenchyma enhancing. We excluded the trachea and blood vessels from the lung to obtain a healthy lung parenchyma area. We further determined the position and width of the optimal window by calculating the median and standard deviation of the healthy lung parenchyma CT signal, which is generally used for observing lung parenchymal lesions. Finally, enhanced CT images, namely DLP-enhanced CTs, are obtained as parenchyma abnormalities are significantly enhanced compared to pulmonary windows.

(III) Segment the abnormalities in the lung parenchyma. We compared the enhanced parenchyma with the lung window. As the lesions were enhanced dozens of times, more previously neglected lesions were identified. Based on the DLP-enhanced CT, we built a 2.5D segmentation and quantization model which produced visible and sub-visual COVID-19 lesions from DLP-enhanced CT images. For simplicity reasons, the complete algorithm workflow was called the DLPE method.

#### 2.3.3. Quantitative analysis

We used lung parenchyma lesion volume ratio and median lesion severity to measure the lesion severity of the CT images after DLPE process. The lesion volume ratio is defined as lesion volume divided by lung parenchymal volume:


(1) $$\begin{array}{l} Lesion\;volume\;ratio=V_{subvisual}÷V_{lung} \end{array}$$

Where *V*_*subvisual*_ is the volume of the sub-visual lesions, *V*_*lung*_ is the volume of the lung parenchyma. Median lesion severity is the median value of the difference between the lesion and the baseline CT signal:


(2) $$\begin{array}{l} \begin{array}{l} Median\;lesion\;severity=|Subvisual\;array-Baseline\;array\;| \end{array} \end{array}$$

Where *Baselinearray* is the median CT signal value of healthy lung parenchyma, and *Subvisualarray* is the CT signal value of lesion. Absorption ratio was used to describe the lesion changes from discharge to 6-month follow-up:


(3) $$\begin{array}{l} Absorption\;ratio=\left(Discharged-Followup\right)÷Discharged \end{array}$$

Where *Discharged* represents the lesion volume ratio of hospital discharge CT, and *Followup* represents the lesion volume ratio of follow-up CT. An absorption ratio >0 reveals that the lung lesions have been absorbed since hospital discharge. As such, higher absorption ratios indicate better recovery. Vice versa, an absorption ratio less than or equal to 0 implies that the lung lesions of the patient have enlarged or remained unchanged since hospital discharge. In this case, higher absorption ratios indicate worse recovery; namely, patients may be affected to varying degrees by sequelae such as pulmonary fibrosis.

### 2.4. Statistical analysis

Statistical analyses were performed using Python 3.7. Without otherwise statement, measurement data were described by mean ± standard deviation or median (interquartile range). The Mann-Whitney U test and Kruskal-Wallis test were used to test independent samples. Count data were expressed as frequencies with percentages. *P* < 0.05 was considered to be statistically significant.

## 3. Results

### 3.1. Clinical characteristics

We retrospectively analyzed the clinical data of 41 follow-up patients. Clinical characteristics are shown in Table 1. The mean age of the patients was 42.2 ± 17.7 years, of which 13 were male patients (31.7%) and 28 were female patients (68.3%). The mean BMI of the patients was 25.2 ± 4.4 Kg/m^2^. And there are 14.6% of patients meanwhile suffering from hypertension and 9.8% from diabetes. In the 6 months after discharge, some patients developed sequelae, which including: impaired sleep quality (17.1%), memory decline (26.8%), anxiety (21.9%), depression (4.9%), throat discomfort (9.8%), decline of vision acuity (12.2%), fatigue (7.3%), limbs weakness (4.9%), muscle or joint aches (12.2%) and hair loss (9.8%). None of the patients developed pulmonary-related sequelae such as dyspnea. We investigated the SGRQ scores of follow-up patients. The median of SGRQ scores was 0.041 and the interquartile range was (0, 0.104). All patients had SGRQ scores <1.

**Table 1:** Demographic and clinical characteristics of the enrolled COVID-19 patients.

**Characteristics**	**All patients (*n* = 41)**
Age, years	42.2 ± 17.7
**Sex**	
Men	13 (31.7%)
Women	28 (68.3%)
BMI	25.2 ± 4.4
**Basic diseases**	
Hypertension	6 (14.6%)
Diabetes	4 (9.8%)
**Sequelae**	
Impaired sleep quality	7 (17.1%)
Memory decline	11 (26.8%)
Anxiety	9 (21.9%)
Depression	2 (4.9%)
Throat discomfort	4 (9.8%)
Decline of visual acuity	5 (12.2%)
Fatigue	3 (7.3%)
Arm Weakness	2 (4.9%)
Muscle or joint pain	5 (12.2%)
Hair loss	4 (9.8%)
SGRQ score	0.041 (0, 0.104)

### 3.2. Chest CT evaluation

#### 3.2.1. Imaging evaluation

**Figure 3:**
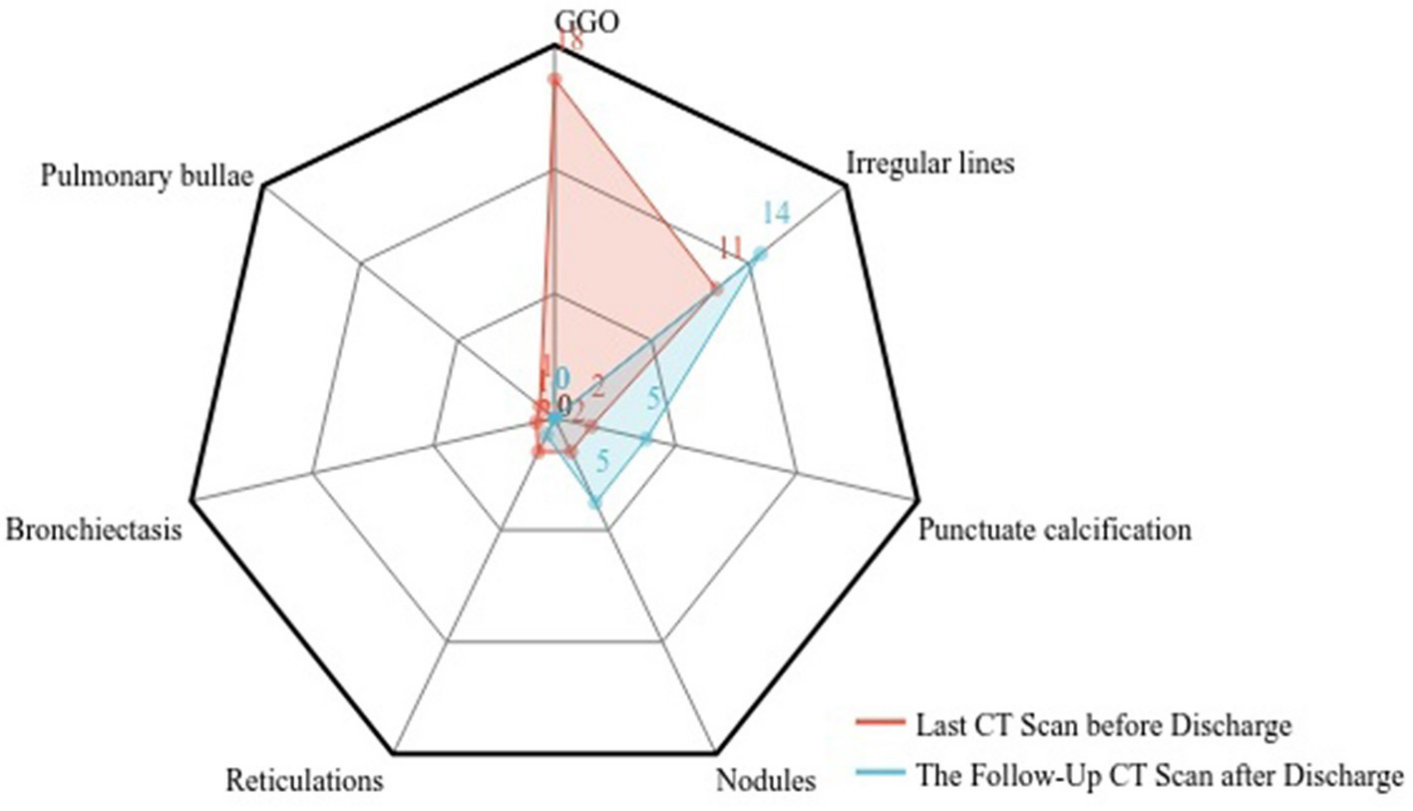
Imaging features of follow-up CT and hospital discharge CT.

**Figure 4:**
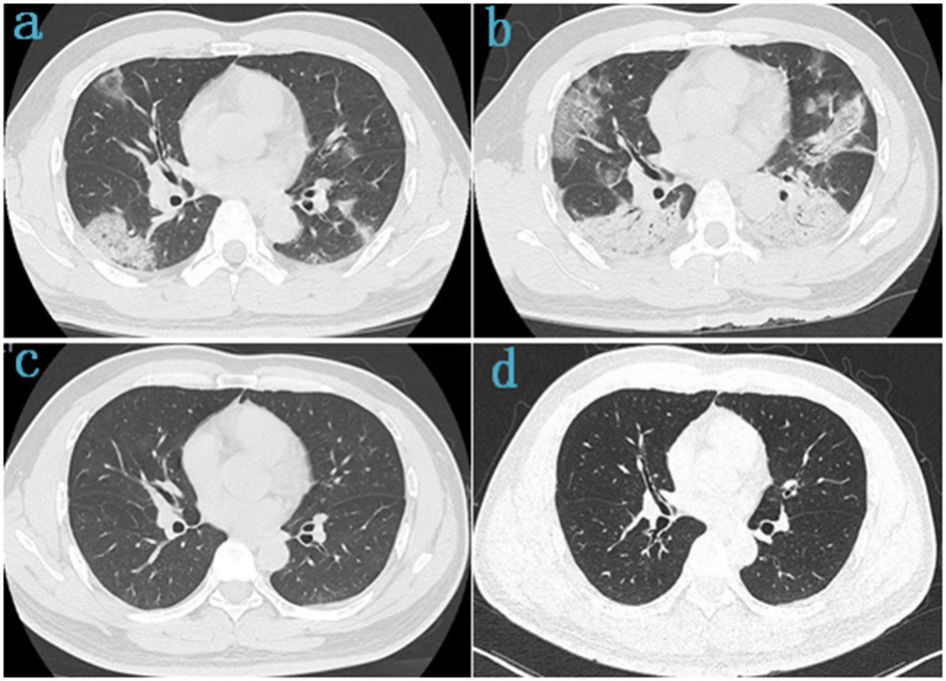
Chest CT of a 42-year-old male survivor of COVID-19 Delta variant. **(a)** On admission, baseline scan shows multiple bilateral ground-glass opacity with predominantly linear pattern and peripheral distribution, with air-bronchogram and tubular size increase of vessels in some lesions. **(b)** 4 days after admission, the lesions were significantly larger and more extensive than before, chest CT scans were subpleural ground-glass opacity that grew larger with crazy-paving pattern and consolidation. **(c)** Before discharge, the lesions in both lungs were basically absorbed. **(d)** At follow-up, the chest CT was basically normal, with a few fibrotic lesions were seen in the left lung.

This study collected the last CT scan before discharge and the 6-month follow-up CT scan from the 41 COVID-19 Delta variant survivors. Two experienced senior radiologists diagnosed all CT scans and summarized the imaging features. As shown in Figure 3, prominent abnormalities found on the CT before discharge include ground-glass opacity in 18 cases (43.9%) and irregular lines in 11 cases (26.8%). A few had punctuated calcification (4.9%), small 201 nodules (4.9%), reticulations (4.9%), and traction bronchiectasis (2.4%). In comparison, the ground-glass opacity was almost utterly unseen in the follow-up CT. Other present abnormalities include irregular lines in 14 cases (34.1%), punctuate calcification in five cases (12.2%), and small nodules in 5 cases (12.2%). Figure 4 demonstrates the lung recovery process of a typical COVID-19 Delta variant survivor.

#### 3.2.2. CT slices after deep lung parenchyma enhancing

**Figure 5:**
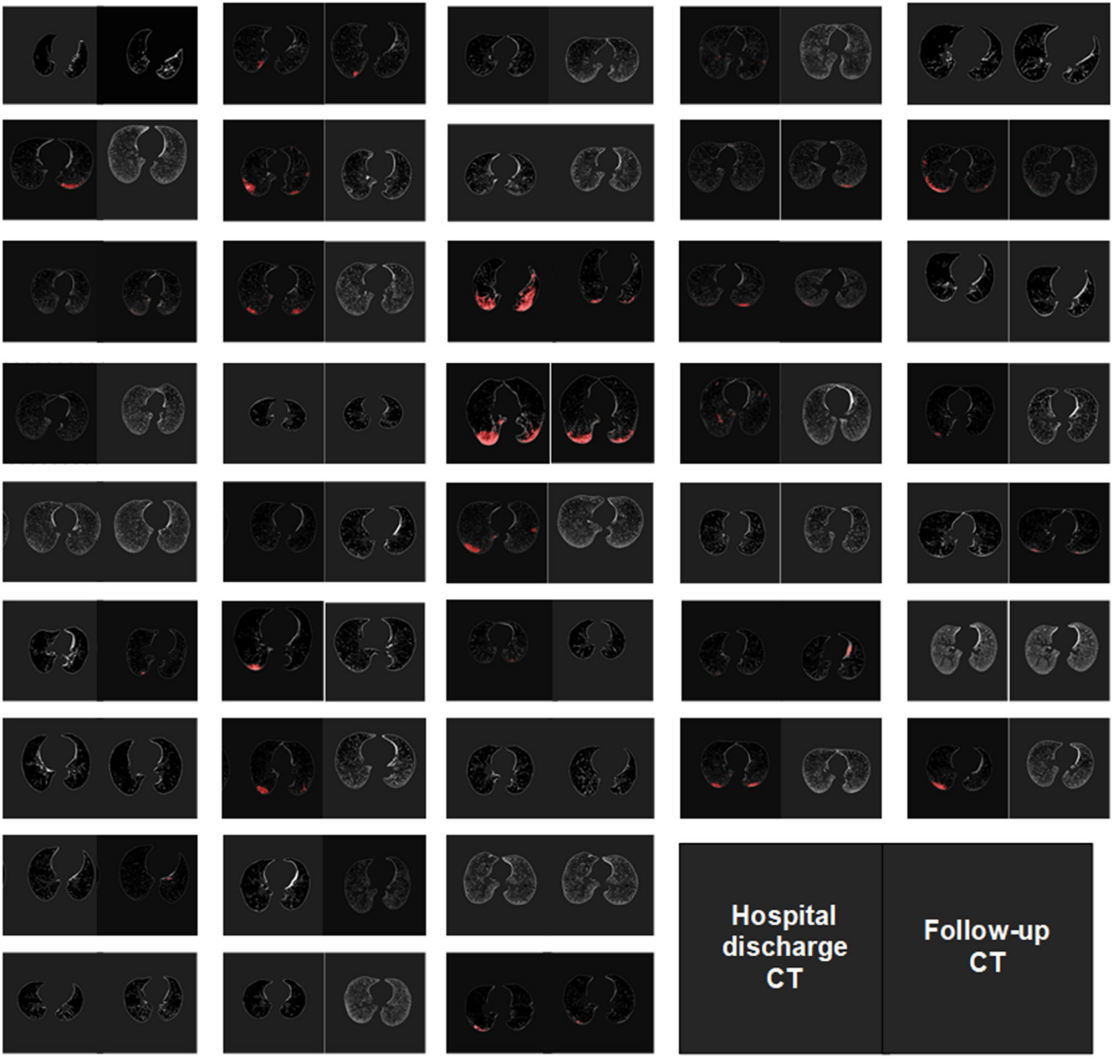
DLPE method applications in 41 patients.

**Figure 6:**
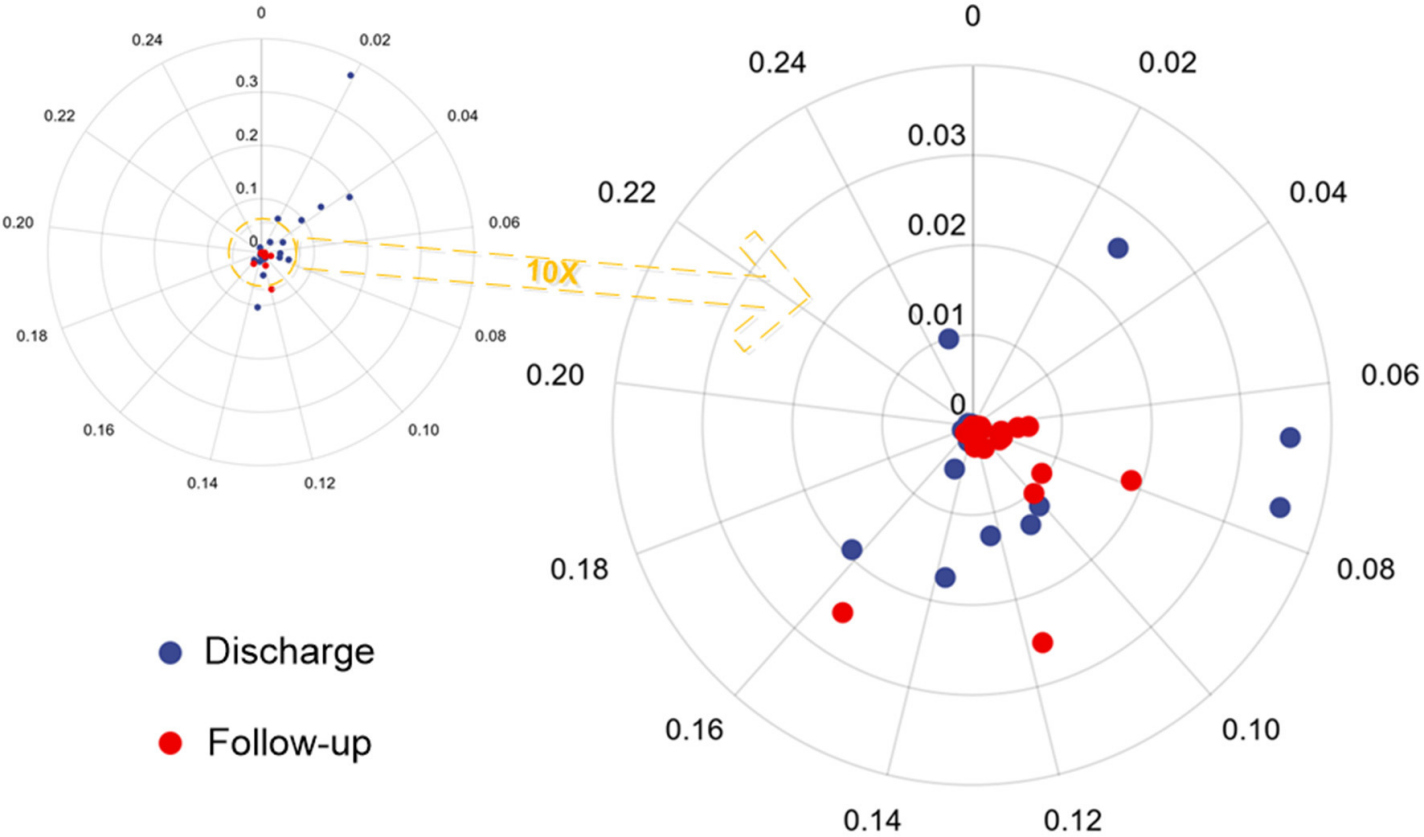
Polar scatter plot based on the lesion volume ratio and median lesion severity from hospital discharge and follow-up CT. The image on the left becomes the image on the right after enlarging 10 times.

We enhanced all CT scans using the DLPE method to visualize all lesions, including sub-visual abnormalities. The comparison between the processed discharged CT and the processed follow-up CT (typical CT slices) shows that most of the lesions had been absorbed by the discharge, and the lung condition had improved considerably in 6-month (Figure 5). In addition, we measured the severity of the detected lesion using the lesion volume ratio and median lesion severity (Table 2). Of note, the lesion volume ratio and median lesion severity were significantly smaller at follow-up than at discharge (*p* < 0.05; Figure 6).

**Table 2:** Changes in lesions under DLPE.

	**The hospital discharge CT (*n* = 41)**	**The follow-up CT (*n* = 41)**	***P*-value**
The lesion volume ratio	0.0018 (0.0003, 0.0353)	0.0004 (0, 0.0032)	0.005
Median lesion severity	0.1329 (0.0632, 0.1892)	0.0910 (0.0730, 0.1179)	0.012

Data are medians, with ranges of quartiles in parentheses.

#### 3.2.3. Absorption ratio at the 6-month follow-up

The mean value of the survivors' absorption ratio was 0.7147 (–1.0303, 0.9945). We grouped patients by gender, age, BMI, and COVID vaccination status, upon which we performed statistical analysis on the absorption ratio concerning median lesion severity (Table 3). While slight statistical difference was seen in the absorption ratio among patients in different BMI range groups (*p* = 0.155), the difference was stronger among different age groups (*p* < 0.005). No significant differences were seen among different gender groups or vaccination status groups.

**Table 3:** Absorption ration in lesions under DLPE.

	**Absorption rate of lesion volume ratio (*n* = 41)**	***P*-value**	**Absorption rate of median lesion severity (*n* = 41)**	***P*-value**
Sex		0.575		0.327
Male (*n* = 13)	0.7147 (–1.3699, 0.9669)		0.4003 (–0.1075, 0.6171)	
Female (*n* = 28)	0.7518 (–0.7423, 0.9981)		0.2354 (–1.8101, 0.5783)	
Age range		0.005		0.302
≤45 (*n* = 22)	–0.7425 (–6.1833, 0.8158)		0.4624 (0.0100, 0.5805)	
45–59 (*n* = 12)	0.9864 (0.3432, 0.9994)		0.0468 (–2.1080, 0.5253)	
≥60 (*n* = 7)	0.9890 (0.7147, 1.0000)		0.3092 (–1.1145, 1.0000)	
BMI		0.155		0.806
≤18.5 (*n* = 4)	0.9059 (–0.5578, 0.9997)		0.3943 (–0.5121, 0.8686)	
18.5–23.9 (*n* = 12)	0.5655 (–1.0708, 0.9880)		0.1803 (–0.2579, 0.5757)	
≥24 (*n* = 18)	0.8694 (–0.1381, 0.9997)		0.4271 (–0.5965, 05846)	
≥28 (*n* = 7)	–0.6000 (–10.746, 0.7147)		0.3059 (–2.4956, 0.5836)	
Vaccinated or not		0.626		0.291
Not (*n* = 15)	0.8238 (–1.0148, 0.9988)		0.3059 (–0.7877, 0.4709)	
Vaccinated (*n* = 26)	0.7077 (–1.0542, 0.9934)		0.4464 (–0.1467, 0.5847)	

Data are medians, with ranges of quartiles in parentheses.

### 3.3. Comparison with original COVID-19 follow-up

We compared the results of this follow-up study with those of the five original COVID-19 follow-up studies, as presented in Table 4. We found that Delta variant survivors had similar sequelae as the original COVID-19 survivors except for severe pulmonary sequelae such as chest tightness and dyspnoea. Further, we confirmed the absence of ground-glass opacity (GGO) and the mild fibrosis of lung lesions in the follow-up CT scans, suggesting that the lung prognosis of Delta variant patients is better than that of the original COVID-19 patients. Specifically, in CT imaging, 62–90% of original COVID-19 patients were discharged with GGO and 7.3–68% had consolidation. However, in our study, only 43.9% of Delta variant patients were discharged with GGO and without consolidation. And at 6-month follow-up, 27–44.8% of original COVID-19 patients still had GGO, while no GGO was found in Delta variant survivors.

**Table 4:** Comparing the original COVID-19 follow-up to the Delta variant follow-up.

**References**	**Sample size**	**Age**	**Basic diseases**	**Sequelae**	**The hospital discharge CT**	**The follow-up CT**
Dai et al. (13)	50	48 ± 14	Hypertension (18%), Diabetes (16%), Pulmonary disease (4%)	Decreased activity tolerance (18%), Cough (10%), Palpitation (6%), Depression (12.5%)	GGO (90%), Consolidation (54%), Reticulations (13%), Bronchiectasis (10%)	GGO (42%), Consolidation (20%), Bronchiectasis (6%), Reticulations (11%)
Han et al. (14)	114	54 ± 12	Hypertension (28%), Diabetes (11%), Chronic pulmonary (14%)	Dry cough (6.1%), Dyspnea (14%), Expectoration (10%)	GGO (62%), Consolidation (24%), Reticulations (14%)	Normal CT (38%), GGO (27%) Fibrotic-like changes (35%)
Jia et al. (15)	205	56 ± 12	Hypertension (36.9%), Diabetes (16%)	-	GGO (87%), Consolidation (7.3%), Reticulation (5.4%), Bronchiectasis (5.4%), Nodule (1%)	Normal CT (48.3%), GGO (28%), Consolidation (1.5%), Reticulation (22%), Bronchiectasis (13.7%), Nodule (7.3%)
Huang et al. (16)	1,733	57 (47–65)	Hypertension (29%), Diabetes (12%), Pulmonary disease (2%)	Fatigue or muscle weakness (63%), Sleep difficulties (26%), Hair loss (22%), Smell disorder (11%), Palpitations (9%), Anxiety or depression (23%)	GGO (76%), Consolidation (23%), Irregular lines (30%)	Normal CT (47.3%), GGO (44.8%), Consolidation (1.1%), Irregular lines (15.9%)
Caruso et al. (17)	118	65 ± 12	Hypertension (34%), Diabetes (9.0%)	Cough (24%), Dyspnea (42%), Hair loss (20%), Decline of visual acuity (12%)	GGO (86%), Consolidation (68%), Fibrotic-like changes (55%)	Normal CT (28%), GGO (42%), Consolidation (1.7%), Fibrotic-like changes (72%)
Our study	41	42.2 ± 17.7	Hypertension (14.6%), Diabetes (9.8%)	Impaired sleep quality (17.1%), Memory decline (26.8%), Anxiety or depression (26.8%), Fatigue (7.3%), Hair loss (9.8%)	Normal CT (34.1%), GGO (43.9%), Fibrotic-like changes (34.1%)	Normal CT (51.2%), GGO (0%), Fibrotic-like changes (48.8%)

## 4. Discussion

Pulmonary fibrosis is an interstitial lung disease caused by intense fibroblast activation and extracellular matrix deposition in the lung, which often results in a range of sequelae such as reduced diffusion function of lung and labor dyspnea in patients. To date, studies on the follow-up of original COVID-19 patients have found that the most common abnormal lung changes in discharged patients are fibrosis and ground-glass opacity (GGO) (16, 18–20), consistent with SARS-related research foundings. Studies have shown that a shell nucleoprotein from SARS can bind to SMAD3, a cellular protein that activates a signaling pathway to promote collagen and plasminogen protein inhibitor production, further leading to the formation of fibrosis in the lungs (21). At present, no similar protein has been identified in COVID-19-related studies, so the current understanding of the prognosis of fibrotic changes in COVID-19 patients remains unclear. Caruso et al. (17) reported that residual GGO was found on lung CT in 42% of original COVID-19 patients and fibrotic changes were present in 72%; a proportion of patients were discharged with dry cough (24%), dyspnoea (42%) and many other lung-related sequelae. In a 6-month follow-up study of 114 original COVID-19 patients, Han et al. (14) found that 35% of patients had residual fibrotic changes in lungs and 14% had dyspnoea. Besides, seriously ill hospital patients developed more severe fibrosis, which was found to restain even at the 1-year follow-up. Similar results have been seen in a 15-year follow-up study of SARS patients (22). Pan et al. (23) found that 61% of patients had complete resolution of abnormal lung changes by 3 months after discharge; at the 1-year follow-up, 25% of patients still had residual fibrotic changes, but it was unclear whether this fibrosis can be further absorbed.

There are few follow-up studies on the Delta variant; hence we conducted this study to raise awareness of pulmonary fibrotic changes and other COVID-19 Delta variant sequelae. We collected hospital discharge CT scans and 6-month follow-up CT scans from 41 Delta variant survivors. We found that more than half of the patients (51.2%) had no residual fibrosis in their follow-up CT of lung after 6-month discharge and that the GGO was almost completely absorbed. The changes of fibrotic presented in follow-up CT of the remaining patients were predominantly irregular lines (34.1%) and small nodules (12.2%), and the patients had a very mild degree of fibrosis. Artificial intelligence and deep learning techniques are widely adopted in current radiology research as they enable physicians to segment infected lesions accurately and implement precision medicine. This research used the previously proposed deep lung parenchyma enhancing (DLPE) model (7) to automatically outline all lung lesions, including conventional and sub-visual lesions. Quantitative assessments was further conducted to evaluate patients' recovery, comparing the calculations of lung lesions on discharged CT and follow-up CTs. We found that most patients had largely dissipated lung lesions at discharge. And after 6 months, re-quantification of lung lesions on follow-up CT revealed a small lesion volume ratio (mean = 0.04%), leading us to assume that the lung fibrosis had been slowly absorbed over time. In the meantime, a proportion of patients developed increased fibrosis (i.e., negative absorption ratio), yet the observed fibrosis levels were less notable and the pulmonary diffusion function remained unaffected. This suggests that DLPE might be deficient in capturing some existing lung damage (early lung damage). It is also possible that the patients experienced other lung damage after discharge, which also caused fibrosis but was unrelated to the COVID-19 infection.

There are multiple factors that affect the prognosis of COVID-19 survivors, including gender, age, body mass index (BMI), and vaccination status. Sylvester et al. (24) have shown that female patients are at a higher risk of developing long COVID syndrome due to differences in the immune system between the sexes. Obesity, measured by BMI, is considered a risk factor strongly associated with the severity of COVID-19 infection and mortality (25). Vaccination is vital in preventing and treating COVID-19 (26), as it reduces the risk of hospitalization and severe sequelae after infection. Age is also strongly associated with patient prognosis, as Huang et al. (16) found that a 10-year increase in age of COVID-19 patients was associated with a 27% increase in pulmonary diffusion dysfunction and a 4% decrease in the absorption ratio. Results of comparing the absorption ratio by gender, age, BMI, and vaccination status show that the absorption rate of lesion volume ratio was significantly different for different age groups (*p* < 0.005) and slightly different for different BMI range groups (*p* = 0.155). No differences were seen between gender and vaccination status groups, presumably due to the small sample size. In addition, sequelae such as impaired sleep quality, memory loss and anxiety were found in Delta variant survivors, similar to those noted in the follow-up study of 1,733 original COVID-19 patients by Huang et al. (16).

This study used the St. George's Respiratory Questionnaire (SGRQ) (10) to assess the Delta variant survivors and obtained a median SGRQ of 0.041 (0, 0.104), which is within the normal range. The result confirmed that the participating survivors had good pulmonary recovery with no significant pulmonary sequelae, and the infection did not significantly affect their quality of life.

The main contributions of this paper are as follow. 1) This is a 6-month follow-up study on discharged COVID-19 Delta variant patients, with data gathered from an earlier cohort of Delta variant patients in China. As limited follow-up studies have been done on the Delta variant, this research is prevailing in broadening the understanding of the Delta variant. 2) Multiple computer-aided techniques, such as deep learning and quantitative analysis, are used to compare the follow-up outcomes of Delta variant survivors and original COVID-19 survivors. Critical findings include Delta variant survivors had a better prognosis than original COVID-19 survivors. 3) In this study, we applied the previously proposed sub-visual lesion observation method (i.e., DLPE) for the first time. This novel lesion segmentation method enabled clinicians to observe and analyze lung lesion changes in Delta variant survivors in greater detail, which is of great value and guidance for the COVID-19 prognostic assessment. Indeed, this research has some shortcomings. Firstly, we lacked direct information showing the patients' pulmonary diffusion functions because the selected cohort did not undergo a complete pulmonary function test during hospitalization and follow-up examination. Secondly, this study was geared toward Delta variant survivors diagnosed in Zhangjiajie city, China, in 2021, resulting in a small sample size.

## 5. Conclusion

In this study, we analyzed the discharged CT scans, 6-month follow-up CT scans, and some clinical indicators of 41 COVID-19 Delta variant survivors to assess fibrosis absorption and sequelae comprehensively. This paper marked the first application of the deep lung parenchyma enhancing method to quantify the extent of lung lesions on hospital discharge and follow-up CTs. We found that the lung lesions had primarily dissipated by discharge and that the lesion volume ratio in follow-up CT was generally small in most cases. We also compared the absorption ratios by gender, age, BMI, and vaccination status. Results have shown that the absorption ratios were significantly different for patients in different age groups and slightly different for different BMI range groups. Statistics and analysis of Delta variant sequelae are also provided, pointing out the primarily experienced sequelae, including impaired sleep quality, memory loss, and anxiety. In conclusion, this study aims to use computer-aided AI methods to raise awareness of the COVID-19 Delta variant and promote the prognosis of the disease. While confounding progress has been made in understanding pulmonary sequelae associated with the Delta variant, it is absolutely necessary to carry on the investigation of COVID-19 and the evolution of prognosis clinical care continuously in the future.

## Data availability statement

The raw data supporting the conclusions of this article will be made available by the authors, without undue reservation.

## Ethics statement

The studies involving human participants were reviewed and approved by Medical Ethics Committee of Zhangjiajie City People's Hospital. The patients/participants provided their written informed consent to participate in this study. Written informed consent was obtained from the individual(s) for the publication of any potentially identifiable images or data included in this article.

## Author contributions

ML and ZQ conceived this study. JH, ZSu, MZ, HL, CC, and MX collected all the chest CT scans and clinical data. RL, NB, and HL were the developers of computer-aided diagnosis methods. RL and NB completed the data analysis. JH, ZSu, and MZ wrote about the imaging findings of patients. JH, RL, NB, and ZSh drafted the manuscript. All authors were involved in the finalization of the manuscript and approved the manuscript.
